# Evaluation of an impedance-based method to monitor the insertion of the electrode array during cochlear implantation

**DOI:** 10.1007/s00405-024-08584-2

**Published:** 2024-04-02

**Authors:** Timo M. Gottfried, Pablo Galeazzi, Aline Föger, Daniel Dejaco, Andrea Tröger, Natalie Fischer, Veronika Innerhofer, Francesco Di Trapani, Nora Weiss, Josef Seebacher, Angelika Dierker, Joachim Schmutzhard

**Affiliations:** 1 Department of Otorhinolaryngology, Head and Neck Surgery, Medical University of Innsbruck, Anichstr. 35, 6020 Innsbruck, Tyrol Austria; 2 MED-EL Medical Electronics, Worldwide Headquarters, Fürstenweg 77a, Innsbruck, Tyrol Austria; 3 Department of Otorhinolaryngology, Head and Neck Surgery, Medical University of Bochum, North Rhine-Westphalia, Bleichstraße 15, 44787 Bochum, Germany; 4 Department of Otorhinolaryngology, TUM School of Medicine, Klinikum rechts der Isar, Technical University of Munich, Munich, Germany; 5 Department of Translational Neurosciences, Faculty of Medicine and Health Sciences, University of Antwerp, Antwerp, Belgium; 6 Departement of Hearing, Speech and Voice Disorders, Medical University of Innsbruck, Anichstr. 35, 6020 Innsbruck, Tyrol Austria

**Keywords:** Cochlear implant, Electrode impedance, Insertion speed, Real-time measurement

## Abstract

**Purpose:**

Cochlear implantation is a prevalent remedy for severe-to-profound hearing loss. Optimising outcomes and hearing preservation, and minimising insertion trauma, require precise electrode placement. Objective monitoring during the insertion process can provide valuable insights and enhance surgical precision. This study assesses the feasibility and performance of an impedance-based method for monitoring electrode insertion, compared to the surgeon’s feedback.

**Methods:**

The study utilised the Insertion Monitoring Tool (IMT) research software, allowing for real-time measurement of impedance and evoked compound action potential (eCAP) during electrode insertion in 20 patient implantations. This enabled an impedance-based method to continuously assess the status of each electrode during the insertion process. The feasibility and performance was evaluated and compared to the surgeon’s feedback approach. eCAP measurements focused merely on feasibility without searching specific responses.

**Results:**

The IMT demonstrated feasibility in measuring real-time impedances and eCAP during the insertion of the electrode array. The impedance-based method exhibited potential for accurately monitoring the insertion depth with a high success rate. However, further development is needed to improve the number of usable contacts.

**Conclusions:**

Objective monitoring with the impedance-based method shows promise as a valuable tool to enhance the precision of cochlear implant electrode insertion respecting insertion distance estimation. The IMT research software proved feasible in recording real-time impedances and eCAP during electrode insertion. While this impedance-based method exhibits high success rates, further improvements are required to optimise the number of usable contacts. This study highlights the potential of objective monitoring techniques to enhance cochlear implantation outcomes.

## Introduction

Cochlear implantation is a well-established treatment for severe-to-profound hearing loss in both adults and children [1, 2]. However, achieving optimal outcomes in hearing preservation depends on various factors, including electrode placement and minimising insertion trauma [3, 4]. While there are theoretical approaches to reduce insertion trauma, recent studies and guidelines emphasise the importance of preserving all inner ear structures during implantation [2, 5–8]. To ensure optimal electrode placement and minimise insertion trauma, it is crucial to monitor the insertion process. Currently, surgeons’ visual feedback—and communicating it verbally if necessary—is the most common monitoring method [9], but objective ways to monitor the insertion process could be beneficial in several areas. For example, in cases where hearing preservation techniques involve partial insertion of an electrode array [10], objective monitoring of the insertion process can help to achieve optimal electrode placement. Additionally, in cases where electrocochleography measurements are intended to be performed at a certain distance or angle from the cochlea, access as would be needed for a robotic-assisted cochlear implant array insertion system, as presented by A. Henslee and colleagues [11] for example, objective monitoring could provide the surgeon with valuable information to find this position. Finally, objective monitoring can be especially important in minimal invasive procedures where the electrode insertion cannot be visually inspected [12, 13].

Objective measurements, such as evoked compound action potential (eCAP), impedance and field telemetry (IFT), and evoked stapedius reflex threshold (eSRT), are commonly used during cochlear implantation surgery to verify correct function and placement of the implant [14]. The potential of impedance measurements regarding intracochlear electrode placement has already been discussed in a study concerning 22 implantations by Pile and colleagues [15]. Although these measurements have the potential to monitor the insertion of the electrode array, a break is needed in the insertion process, since they require a considerable amount of time. To enable real-time monitoring without pausing the insertion, objective measurements would need to be performed continuously throughout the insertion process. In this study, research software that measures IFT and eCAP continuously during insertion has been used to study an impedance-based method with potential eCAP functionality for determining the status of an electrode contact during insertion of the electrode array.

The main goal of this study was to evaluate the feasibility of the developed tool and to evaluate the performance of an impedance-based method for monitoring the insertion process, while comparing it to the commonly used method (surgeon’s feedback). The secondary purpose was to assess whether the study setup is able to measure eCAP values reliably.

## Materials and methods

### Ethics statement

This study conformed to the Declaration of Helsinki and the guidelines of the Institutional Ethics and Research Committee of the Medical University of Innsbruck, which approved the study. Informed consent was obtained from all participants.

### Participants

Between 2021 and 2022, a total of 20 patients (13 males, 7 females) who underwent cochlear implantation from 20 to 88 years of age (mean: 62 years, STD: 17 years) participated in the study. They were all implanted with an MEDEL Synchrony II® Cochlear Implant (CI) (MedEL, Innsbruck, Austria) with Flex electrode array (1 Flex 24, 18 Flex 28, 1 Flex Soft). Eleven patients received a CI on the right side and nine on the left side (Table 1). Each patient underwent a pre-operative and post-operative CT-Scan.

**Table 1:** Study demographics

Case	Sex	Implanted ear	Age	Surgical approach	Electrode type
IK_01	Female	left	61	Previous mastoidectomy	Flex 28
IK_02	Male	left	56	Conventional approach	Flex 28
IK_03	Male	left	58	Conventional approach	Flex Soft
IK_04	Female	right	59	Conventional approach	Flex 28
IK_05	Female	left	30	Conventional approach	Flex 28
IK_06	Male	right	82	Conventional approach	Flex 28
IK_07	Male	left	63	Conventional approach	Flex 24
IK_08	Female	right	61	Conventional approach	Flex 28
IK_09	Male	left	74	Conventional approach	Flex 28
IK_10	Male	left	70	Conventional approach	Flex 28
IK_11	Male	left	64	Conventional approach	Flex 28
IK_12	Male	right	77	Conventional approach	Flex 28
IK_13	Female	right	20	Conventional approach	Flex 28
IK_14	Male	right	75	Conventional approach	Flex 28
IK_15	Male	right	28	Conventional approach	Flex 28
IK_16	Male	left	69	Conventional approach	Flex 28
IK_17	Female	right	72	Conventional approach + tympanoplasty Type I	Flex 28
IK_18	Male	left	62	Conventional approach	Flex 28
IK_19	Male	left	63	Conventional approach	Flex 28
IK_20	Female	right	88	Conventional approach	Flex 28

### Software

**Fig. 1:**
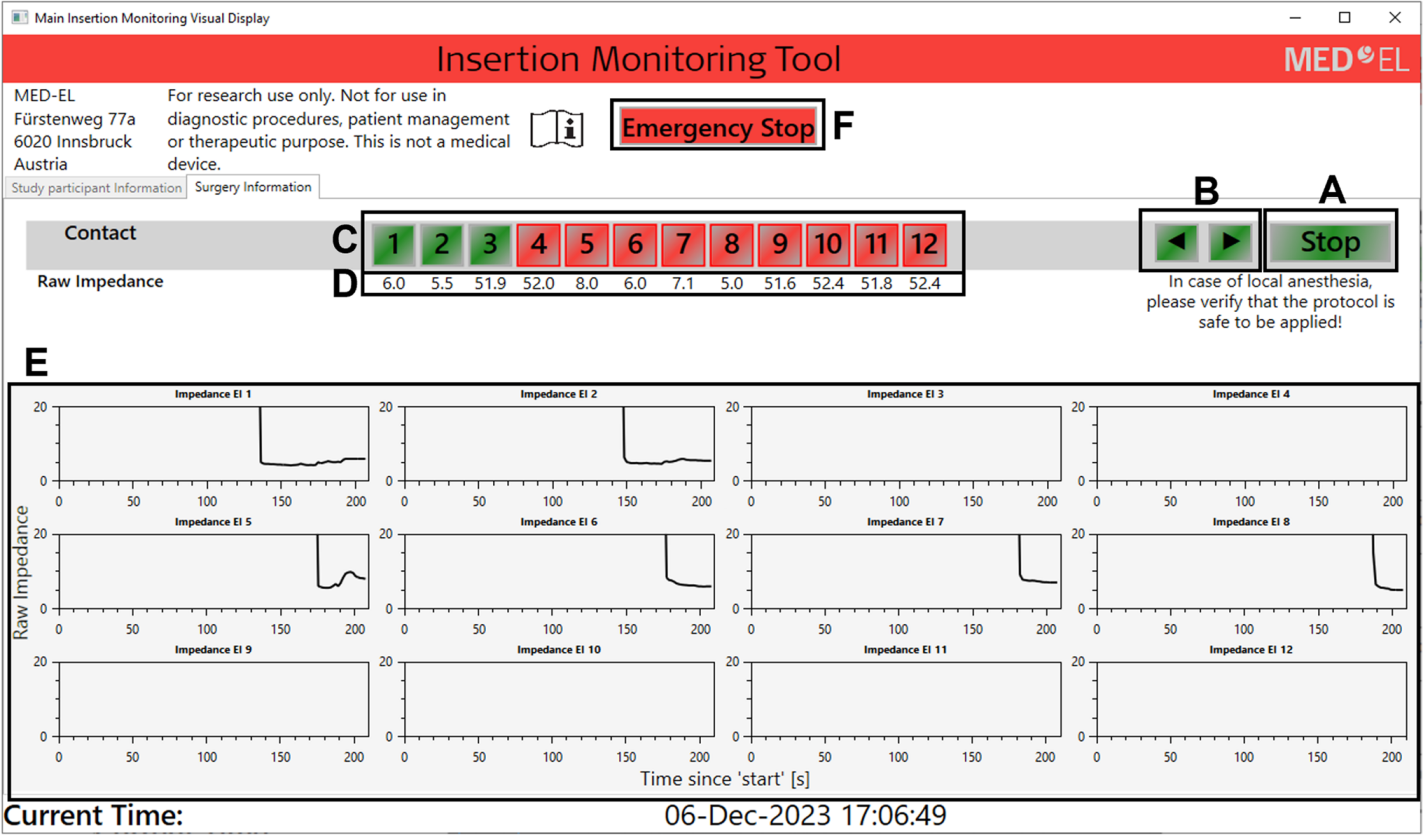
IMT research software GUI. Section **A**: Start and stop button. Section **B**: Next and back arrows allow the user to indicate when an electrode was inserted into the cochlea based on the surgeon’s feedback. Section **C**: Displays the status of each electrode contact based on the surgeon’s feedback. Green: contact inside the cochlea; red: contact outside the cochlea. Section **D**: Displays the current impedance values for each electrode contact. Section **E**: Displays the impedance value through time for each electrode contact. Section **F**: Interrupts the communication with the implant and closes the GUI

The Insertion Monitoring Tool (IMT) research software was developed by MED-EL Medical Electronics and is intended to perform continuous measurements during the insertion of the electrode array in CI surgery, such as Impedance and Field Telemetry (IFT) and evoked Compound Action Potential (eCAP). Figure 1 shows the IMT research software Graphical User Interface (GUI), which allows the user to start the measurement (section A), visualise the IFT measurement (sections D and E), determine manually whether an electrode is inside or outside the cochlea (section B and C) and interrupt the measurements if needed (section F).

The IFT and eCAP values are continuously measured and stored in a local database. In the GUI, measured impedance values are displayed in real time for all 12 contacts (Section C) and updated sequentially in cycles lasting about 1.7 s. Previously measured impedance values are also displayed, in the form of twelve impedance trough time plots (section E).

### Measurement setup

**Fig. 2:**
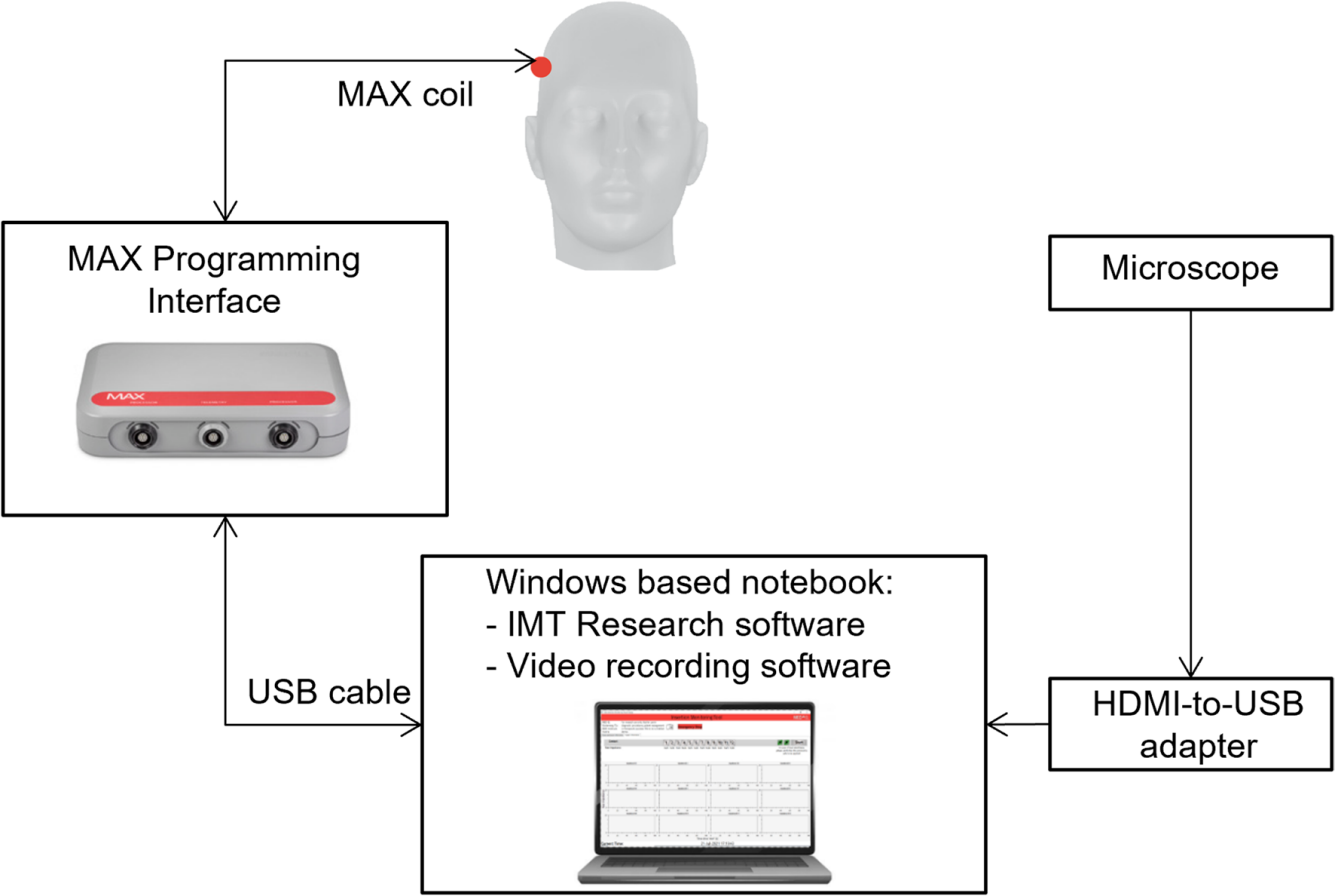
Setup used during the insertion of the array to measure IFT and eCAP in real time

The measurement setup used for the study is shown in Fig. 2. A Windows-based notebook with the IMT research software, an MAX box, and an MAX coil are used to record the electrophysiological measurements during the insertion of the electrode array. In addition, a video output of the microscope is connected to the computer via an HDMI-to-USB converter (Atomos Connect 4 K HDMI-USB Converter). Video recording software (OBS Software, version 26.1.1.) is used to perform a simultaneous recording of the IMT research software and the microscope output.

### Surgical procedure

The surgical technique used was the same in all patients. After pre-operative setup including full anaesthesia plus regional anaesthesia, facial nerve monitoring (Neurosign 100, Neurosign surgical, Whitland, UK) was installed. Via retroauricular incision, the planum mastoideum was visualised and the periosteum was cut into a U-shape and lifted from the mastoid bone. After identifying the linea temporalis and spina suprameatal, a conventional mastoidectomy was performed. An antrotomy was done and the short process of the incus was visualised. The posterior tympanotomy was performed while continuously monitoring the facial nerve. Then, a bone housing was prepared for the implant and a Synchrony II implant was placed, followed by the opening of the round window. By this time, the IMT research software and measurement setup had been already set up as described in Fig. 2. The measurement was started before coupling the MAX coil to the implant, so the correct coupling could be verified, while confirming the data was being measured and displayed. Once the coupling was successful, the surgeons started the insertion of the electrode array. While the electrode was being inserted in the cochlea, the surgeon provided the operator of the IMT research software with feedback on when each electrode contact was being inserted. Once the insertion was completed, the surgeon informed the user of the IMT research software, and the measurement was stopped. Using the MAESTRO software, routine IFT, eCAP, and eSRT were performed, after which the wound was closed.

Post-operative CT-Scans were conducted to confirm the position of the electrode array.

### Feasibility evaluation

The first step of the study involves assessing the feasibility of the IMT research software. The feasibility analysis consisted of two parts:

- *Feasible to measure* The percentage of cases in which it was feasible to set up the system, measure IFT, and measure eCAP, regardless of whether the measurements provided useful results or not.
- *Number of usable contacts* The number of usable contacts was determined from the cases where successful impedance field telemetry (IFT) measurements were obtained. To define a contact as usable, the impedance measurement needed to exhibit a clear drop, indicating whether the contact was located inside or outside the cochlea. This determination of contact usability was performed manually by an experienced clinical scientist. It is important to note that not all contacts consistently showed a clear drop in impedance. Therefore, the usability of each contact was carefully assessed based on the presence of a distinct impedance drop.

### Performance evaluation

The second step of the study focused on the performance of two methods to determine, in real time, the status of an electrode contact, while the electrode array is being inserted into the cochlea. The status of a contact can either be inside or outside the cochlea.

Two different approaches are used to determine the status of each electrode contact:

Method A is based on continuous impedance measurements (impedance-based), whereas Method B uses the assessment of the surgeon during the insertion in real time (surgeon’s verbal feedback).

In addition, a third method, dubbed “video analysis”, was used to determine the insertion time of each contact. This method was used as a ground truth and is referred to as Method C. The usability of contacts, as determined in the feasibility assessment, is also considered in the performance evaluation. For Method A, only the usable contacts are used for the performance evaluation, while, for Method B, the IMT research software will record the surgeon’s feedback for all contacts.

The goal is to compare the performance of the newly developed method (Method A) and the commonly used method (Method B) against the ground truth (Method C).

**Method A** consisted in a retrospective analysis of the usable contacts to determine the insertion time. For each contact, its status was marked for each sample, taking the following requirements into account:

- An electrode contact is “inside” if the impedance is lower than 25 kΩ, or if a more basal contact is “inside” with respect to the same criterion.
- An electrode contact is “outside” if the impedance is equal to or higher than 25 kΩ and all basal contacts are also “outside”.
- The insertion time for an electrode contact is defined as the last time the contact changes from status outside to inside.

**Fig. 3:**
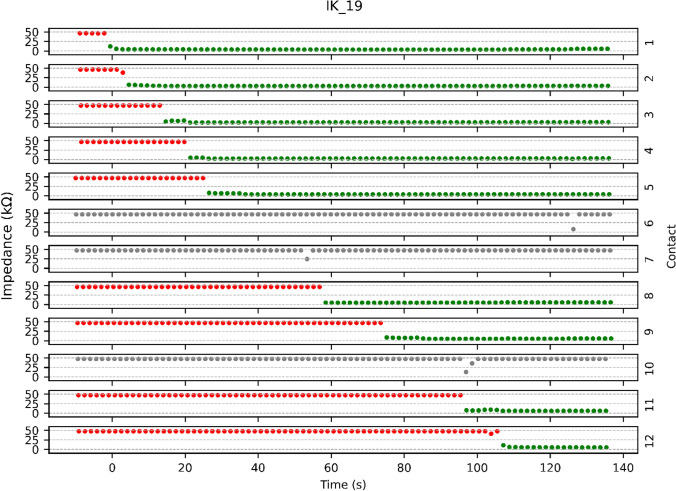
Example of Method A analysis for case IK_19. The graph displays 12 subgraphs, each representing the impedance values over time for a different electrode contact. The x-axis represents the impedance measured in kΩ, while the y-axis represents time in seconds, where x = 0 s refers to the time when the insertion started. As an example of how to interpret the graph, at contact 8, a significant drop in impedance can be observed around 60 s after beginning the insertion of the electrode array. Red dots represent high impedance values, while green dots represent low impedance values. Grey dots indicate a failed measurement

Figure 3 shows an example of Method A for case IK_19. It shows the impedance measures over time for each electrode contact. For contacts 6, 7, and 10, it was not possible to determine the status of the contacts from the respective IFT data; therefore, those contacts are deemed unusable and are marked grey. In the usable contacts, the status of a contact is marked red for outside the cochlea, and green for inside the cochlea.

**Method B** used the surgeon’s feedback during the insertion of the electrode array to determine when each electrode contact was inserted into the cochlea. Section B of Fig. 1 shows how the IMT research software was used to store the feedback provided by the surgeon.

**Method C** (ground truth) consisted in a retrospective analysis of the video recordings, as obtained from the setup described in Fig. 2 Insertion times for all visible contacts have been determined from the video recordings using the annotation software ELAN [16] which makes it possible to create and visualise annotations synchronised with the video timeline. The insertion times were defined as the last time each contact is seen entering the cochlea, consistently with the definition used in Method A.

In addition to insertion of contacts 1–12, the following events were annotated:

- Start of insertion (Annotation: “tip”).
- Stopper at the round window (Annotation: “stopper”).
- End of insertion (Annotation: “end”).

By default, annotations in ELAN are linked to time-intervals of media files and are therefore characterised by both “Begin Time” and “Duration” (or “End Time”) of the annotation. In this work, we chose to align “Begin Time” of annotations with actual insertion times, and to assign arbitrary “Duration” values, which allow for a clear visualisation of the annotations inside the ELAN software. The “Duration” of annotations was only used for visualisation purposes inside the ELAN software and did not represent any value relevant to the analysis.

The performance of the impedance-based method (Method A) and the surgeon’s feedback (Method B) are analysed by comparing them to the ground truth (Method C):

- **Error analysis:** Time difference between the insertion time of an electrode contact for methods A and B compared to the ground truth.
- **Success rate**: For each usable contact, the success rate is defined in this study as the percentage of time in the defined interval (start – end) in which the status obtained from the impedance value is equal to that given by the ground truth. For each case, the success rate is given by the average success rate of all useful contacts.

The performance analysis is conducted from ten seconds before the start of the electrode tip insertion until 10 s after the stopper of the electrode array reaches the round window or the surgeon stops the insertion process.

## Results

### Feasibility evaluation

Table 2 presents the feasibility results of using the IMT research software for each case, indicating whether the tool was successfully set up and whether IFT and eCAP measurements were performed. In 19 out of 20 cases, the tool was successfully set up and IFT and eCAP measurements were performed, indicating a feasibility rate of 95%. In 1 out of 20 cases, the setup of the system failed during the coupling of the MAX coil to the implant, which prevented the measurement of IFT and eCAP.

**Table 2:** Feasibility results

Case	Setup possible	IFT possible	eCAP possible
IK_01	Yes	Yes	Yes
IK_02	Yes	Yes	Yes
IK_03	Yes	Yes	Yes
IK_04	Yes	Yes	Yes
IK_05	Yes	Yes	Yes
IK_06	Yes	Yes	Yes
IK_07	Yes	Yes	Yes
IK_08	Yes	Yes	Yes
IK_09	No	No	No
IK_10	Yes	Yes	Yes
IK_11	Yes	Yes	Yes
IK_12	Yes	Yes	Yes
IK_13	Yes	Yes	Yes
IK_14	Yes	Yes	Yes
IK_15	Yes	Yes	Yes
IK_16	Yes	Yes	Yes
IK_17	Yes	Yes	Yes
IK_18	Yes	Yes	Yes
IK_19	Yes	Yes	Yes
IK_20	Yes	Yes	Yes

Figure 5A illustrates the number of usable contacts for Method A in the 19 cases where the system was successfully set up and IFT and eCAP measurements were performed. In all cases, a range of 2–12 contacts were found to be usable, with an average of 7.9 usable contacts per case. In contrast, as discussed in the performance evaluation section, Method B recorded the surgeon’s feedback for all inserted electrodes, resulting in 12 usable contacts for each case.

Figure 5B illustrates the occurrence of each contact being usable. Each contact was usable in between 9 and 18 cases out of 19, with an average usability of 12.5 cases (66%). However, contacts 11 and 12 exhibited a higher frequency compared to the other contacts. They were usable in 16 out of 19 cases (84%) and 18 out of 19 cases (95%), respectively.

### Performance evaluation

#### Error analysis

**Fig. 4:**
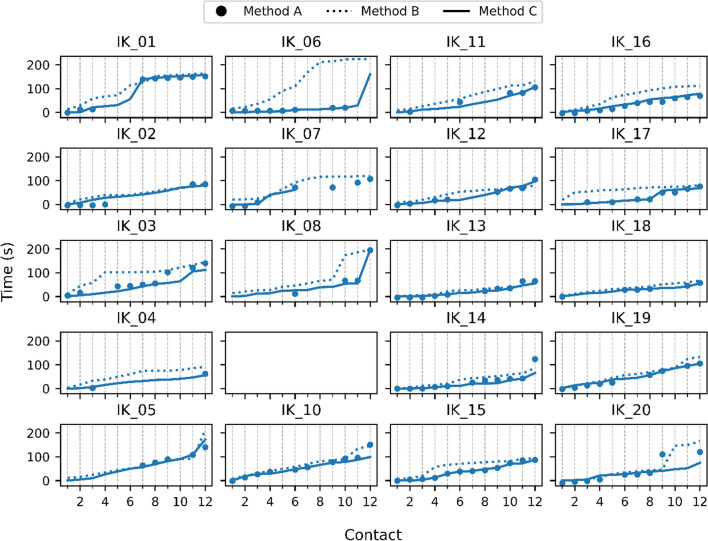
Insertion time for each case and method. Method A (impedance-based) is represented by large dots, Method B (surgeon’s feedback) by small dots, and Method C (ground truth) by solid lines. The x-axis displays the time in seconds, while the y-axis represents the number of contacts along the electrode array

The results of the insertion time for each case and method are displayed in Fig. 4. In case IK_07, it was not possible to determine insertion times for contacts 7–12 using Method C due to limited visibility in the video recording of the microscope. Additionally, Table 2 indicates that the IMT research software failed while setting up the system of case IK_09, which excluded it from the analysis. Therefore, 19 insertions were analysed in the performance evaluation of Methods A and Method B against Method C, where only contacts 1–6 were compared for the insertion of case IK_07.

**Fig. 5:**
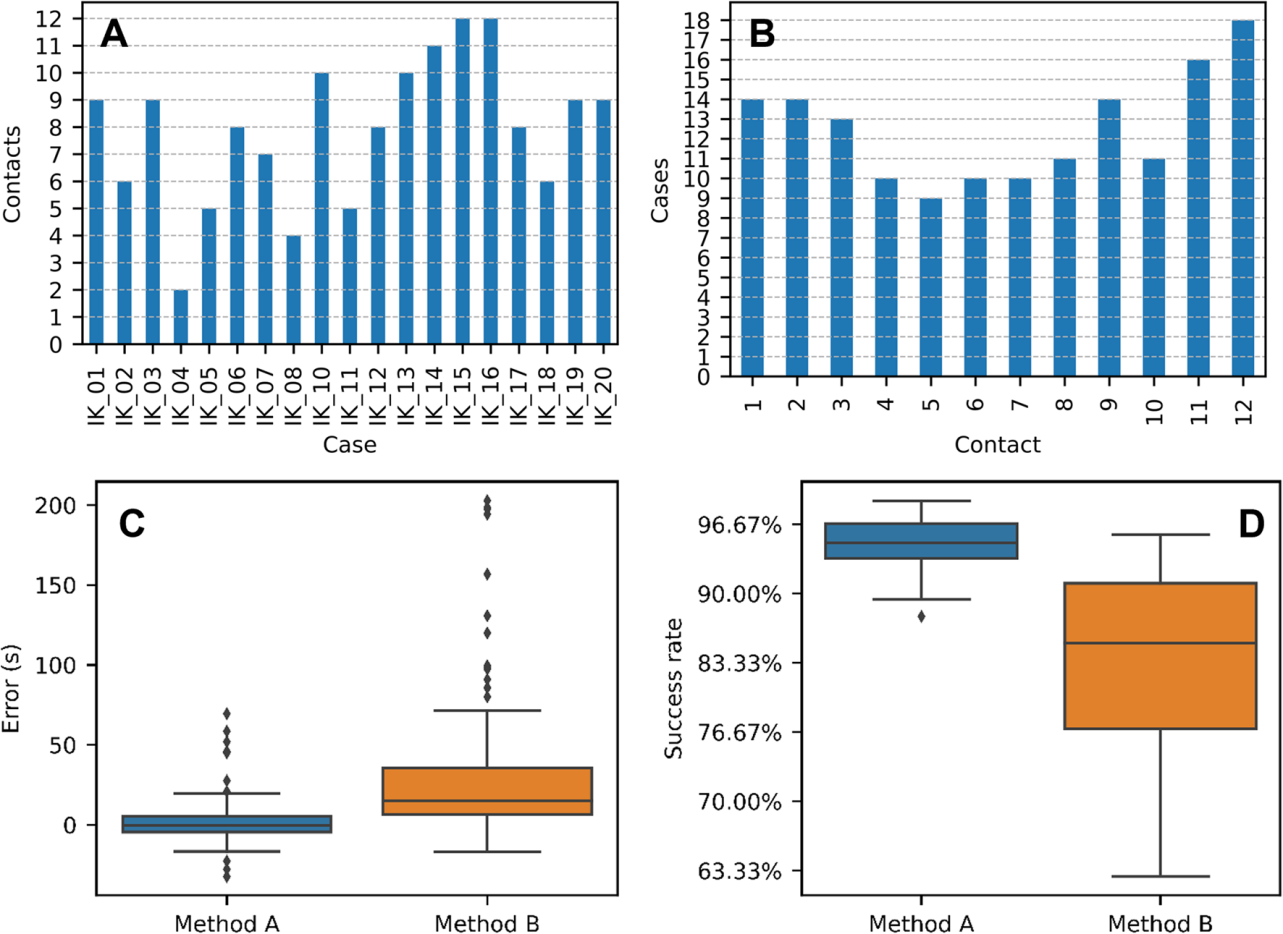
**A–D** A Number of usable contacts per case for Method A. **B** Usability of individual contacts for Method A. **C** Boxplot of the error in time from Method A (impedance-based) and Method B (surgeon’s feedback) compared to method C (ground truth). **D** Boxplot of success rates for Method A (impedance-based) and Method B (surgeon’s feedback) compared to Method C (ground truth)

In Fig. 5C, the boxplot displays the error in seconds for Method A and Method B compared to Method C. The mean error for Method A was 1.66 s with a standard deviation of 13.08 s, and for Method B, it was 25.86 s with a standard deviation of 34.02 s. The differences in error between Method A and Method B were statistically significant with a *p* value of less than 0.001 (Wilcoxon matched pairs signed-rank test).

#### Success rate

Table 3 displays the success rates of Method A and Method B for each case, while Fig. 5D presents a boxplot that illustrates the distribution of the success rates for both methods. The success rates for Method A ranged from 87.80 to 98.95%, with a mean of 94.55% and a standard deviation of 2.93%. On the other hand, the success rates for Method B varied from 62.75 to 95.70%, with a mean of 82.84% and a standard deviation of 9.71%. The differences in success rate between Method A and Method B were statistically significant with a *p* value of less than 0.001 (Wilcoxon matched pairs signed-rank test).

**Table 3:** Comparison between success rates of Methods A and B

Case	Method A (%)	Method B (%)
IK_01	97.22	87.99
IK_02	87.80	93.64
IK_03	90.08	68.41
IK_04	95.26	72.55
IK_05	94.91	95.70
IK_06	98.95	62.75
IK_07	94.20	85.00
IK_08	94.87	82.47
IK_10	94.48	90.91
IK_11	95.22	80.32
IK_12	96.77	87.18
IK_13	92.35	91.75
IK_14	93.28	87.36
IK_15	97.52	79.43
IK_16	93.56	74.57
IK_17	95.15	64.96
IK_18	98.68	91.05
IK_19	96.75	92.71
IK_20	89.46	85.25

## Discussion

This study aimed to assess two aspects: (1) the feasibility of research software designed to monitor the insertion of an electrode array in a CI surgery in real-time and (2) the performance of an impedance-based method in determining the status of each electrode’s array contact during the surgery.

The feasibility assessment showed that the IMT research software was able to successfully measure impedance values and eCAP. Additionally, the GUI was able to visualise the impedance value in real time for each electrode. The eCAP measurements were simplified to confirm their feasibility without employing any specific method, such as the amplitude growth function, to identify an eCAP response. As indicated in Table 2, the measurements were successfully performed in 95% of the cases, indicating a high feasibility rate. These findings suggest that future developments of the IMT research software could prioritise the inclusion of eCAP responses.

The impedance-based method (Method A) developed to determine the status of an electrode contact, while the electrode array is being inserted into the cochlea seems to have a significant improvement compared to the surgeon’s feedback (Method B). However, the relatively lower number of average usable contacts (7.9) for Method A, compared to Method B (12), suggests that there is still potential for enhancing the clinical utility of the impedance-based method. Figure 5A illustrates that in five cases, the number of usable contacts was 50% or less. Potential explanations for this observation include (a) the distance between the MAX coil and the cochlear implant produces a loss of data while measuring, and (b) very small air entrapments cause the lack of drop in the initial real-time impedance measurement. Further investigation is recommended to gain a deeper understanding of the underlying factors contributing to the lower number of usable contacts in Method A.

On the contrary, Fig. 5B highlights the substantial usability rates of 84% and 95% for the last two contacts of the electrode array. These findings suggest a high level of usability in cases where the electrode array is fully inserted, indicating the potential of this method as a confirmation tool for complete electrode array insertion.

However, there is a clear need for further improvement in real-time methods for determining the status of the electrode contacts during the insertion of the electrode array. Tan et al. conducted a study in 2013 to address this issue. The study involved investigating real-time measurements of impedance values in a cadaveric temporal bone experiment, as well as in two live surgeries. Their study focused on utilising impedance measurements to characterise the individual position of each contact within the scala tympani, comparing them to fluoroscopic real-time readings. Similar to our findings, Tan et al. found that real-time impedance values were feasible in determining the insertion status of each electrode contact during the insertion process. However, in contrast to our investigation where all contacts were measured, Tan et al. obtained impedance values for only five individual contacts. They emphasised the need for further system development and procedure standardisation to accurately determine the precise position of the electrode [17].

In 2022, Salkim and colleagues also highlighted the urgent need for an objective real-time method to determine electrode status. They conducted an analysis of impedance variations at different insertion depths in a computational model of the cochlea [7]. Their findings provided additional evidence to support the idea that impedance measurements hold potential as a valuable tool for determining the positioning of the electrode array. This further strengthens the conclusions drawn from our study.

As previously mentioned, the current gold standard for electrode positioning and insertion speed during cochlear implant surgery relies on direct visual control, haptic feedback, and the surgeon’s experience. However, with the increasing focus on minimally invasive surgical techniques for cochlear implantation, such direct visual observation may not no longer be a sustainable gold standard in the future [4, 12, 13]. The results of our study highlight the superiority of the impedance-based method in evaluating the intracochlear location of each contact compared to the gold standard (Fig. 5D). This underscores the need for an objective and reliable replacement. Furthermore, there is a rather high gap between the surgeon’s feedback and the results of the video analysis. This could be explained by the possibility of playing the recorded video fast forward and backward. Naturally, a function missing during the implantation.

In line with this, recent experimental research by Henslee and colleagues developed an electrocochleography-guided robotics-assisted cochlear implant array system in a recent experimental study [11]. Their study suggests that an objective real-time measurement, in their case utilising ECochG, could offer the potential to immediately adjust the robot’s insertion motion and minimise trauma associated with the insertion process. Real-time impedance measurements could serve a similar purpose, assuming a constant insertion speed, with fewer equipment requirements and without the need for residual hearing. However, further studies are necessary to directly compare these two procedures. The findings in this study could facilitate an objective approach to monitor the insertion process of the electrode array during its insertion and therefore help to improve the upcoming robot-assisted minimal approaches.

Furthermore, current practice in minimal invasive procedures often involves the need for a post-operative CT or Cone Beam CT (CBCT) scan to verify the position of the electrode array following a full robotic implantation [13, 18]. However, the utilisation of real-time impedance measurements as a method may provide valuable information to assist the surgeon in determining the current and final position of the electrode array, potentially eliminating the requirement for a post-operative CT or CBCT scan. This could streamline the surgical process and reduce the need for additional imaging.

Lenarz et al. proposed a partial insertion technique of a full-length electrode array in Electric Acoustic Stimulation (EAS) implantation, with the aim of preparing for future advancements in patients’ hearing loss [10]. In this context, the use of an objective method, such as real-time impedances, could assist in optimising the positioning of the electrode lead. Compared to the surgeon’s visual and haptic control, real-time impedances have shown superiority. By utilising this method, the surgeon can precisely insert the intended number of contacts, thereby reducing insertion trauma and preserving residual hearing to the greatest extent possible. This approach aligns with the goal of achieving optimal outcomes in EAS implantation procedures.

In summary, the demonstrated feasibility of the IMT research software in measuring real-time impedances during electrode insertion marks a notable advancement. However, ongoing research is essential to enhance the method’s usability for real-time detection of electrode contacts within the cochlea. Upon achieving heightened usability, the integration of real-time measurements with various impedance-based methods, as proposed by Giardina [19] and the real-time estimation of insertion depth, as emphasised by Aebischer [20], hold promise for predicting array positioning dynamically. Despite the myriad potential applications for this tool, further studies are imperative to substantiate these assumptions and unlock its full potential.

## Conclusion

In conclusion, the results demonstrate the feasibility of the Insertion Monitoring Tool (IMT) research software for real-time recording of impedance values and eCAP during the insertion process. The impedance-based method developed for monitoring the insertion process shows promising accuracy and a high success rate. Nonetheless, further development is required to enhance the number of usable contacts. These findings contribute to the advancement of objective monitoring techniques in cochlear implantation, highlighting the potential for improving surgical outcomes and optimising electrode placement.

## Data Availability

All data regarding this study are available.

## Abbreviations

IMT: Insertion Monitoring Tool
CI: Cochlear Implant
IFT: Impedance Field Telemetry
eCAP: Electrically evoked Compound Action Potential
GUI: Graphical User Interface
eSRT: Electrically evoked Stapedial Reflex Threshold
EAS: Electric Acoustic Stimulation
